# The effect of goal-directed hemodynamic therapy on clinical outcomes in patients undergoing radical cystectomy: a randomized controlled trial

**DOI:** 10.1186/s12871-023-02285-9

**Published:** 2023-10-09

**Authors:** Hyun-Kyu Yoon, Min Hur, Dong Hyuk Kim, Ja Hyeon Ku, Jin-Tae Kim

**Affiliations:** 1 Department of Anesthesiology and Pain Medicine, Seoul National University College of Medicine, Seoul, Republic of Korea; 2 Department of Anesthesiology and Pain Medicine, Seoul National University Hospital, Seoul National University College of Medicine, 101 Daehakro, Jongno-gu, Seoul, 03080 Korea; 3 Department of Anesthesiology and Pain Medicine, Ajou University School of Medicine, Suwon, Republic of Korea; 4 Department of Urology, Seoul National University Hospital, Seoul National University College of Medicine, Seoul, Republic of Korea

**Keywords:** Hemodynamic monitoring, Goal-directed hemodynamic therapy, Radical cystectomy, Postoperative complications

## Abstract

**Background:**

This study investigated the effects of intraoperative goal-directed hemodynamic therapy (GDHT) on postoperative outcomes in patients undergoing open radical cystectomy.

**Methods:**

This prospective, single-center, randomized controlled trial included 82 patients scheduled for open radical cystectomy between September 2018 and November 2021. The GDHT group (n = 39) received the stroke volume index- and cardiac index-based hemodynamic management using advanced hemodynamic monitoring, while the control group (n = 36) received the standard care under the discretion of attending anesthesiologists during surgery. The primary outcome was the incidence of a composite of in-hospital postoperative complications during hospital stays.

**Results:**

A total of 75 patients were included in the final analysis. There was no significant difference in the incidence of in-hospital postoperative complications (28/39 [71.8%] vs. 30/36 [83.3%], risk difference [95% CI], -0.12 [-0.30 to 0.07], *P* = 0.359) between the groups. The amounts of intraoperative fluid administered were similar between the groups (2700 [2175–3250] vs. 2900 [1950–3700] ml, median difference [95% CI] -200 [-875 to 825], *P* = 0.714). The secondary outcomes, including the incidence of seven major postoperative complications, duration of hospital stay, duration of intensive care unit stay, and grade of complications, were comparable between the two groups. Trends in postoperative estimated glomerular filtration rate, serum creatinine, and C-reactive protein did not differ significantly between the two groups.

**Conclusions:**

Intraoperative GDHT did not reduce the incidence of postoperative in-hospital complications during the hospital stay in patients who underwent open radical cystectomy.

**Trial registration:**

This study was registered at http://www.clinicaltrials.gov (Registration number: NCT03505112; date of registration: 23/04/2018).

**Supplementary Information:**

The online version contains supplementary material available at 10.1186/s12871-023-02285-9.

## Background

Radical cystectomy is the standard surgical treatment for invasive bladder cancer [1]. Open cystectomy is a complex surgical procedure involving the removal of the bladder, reproductive organs, and pelvic lymph nodes and the creation of a urinary diversion, which is associated with significant perioperative morbidity and mortality [2, 3]. Various perioperative complications can occur after radical cystectomy, such as substantial blood loss, infections, ileus, wound complications, venous thrombosis, and metabolic disturbances [3–5]. Given that postoperative complications can impact the clinical outcomes of patients after surgery [6, 7], it is imperative for those undergoing radical cystectomy to take measures to mitigate these complications.

Postoperative complications may be associated with an imbalance between oxygen demand and supply and impaired peripheral tissue perfusion [8, 9]. Goal-directed hemodynamic therapy (GDHT) utilizes real-time hemodynamic monitoring to meet the increased oxygen demand during surgery, thereby achieving hemodynamic optimization of end-organ blood flow [10]. Many studies have tested the benefits of GDHT in various clinical settings, showing variable results [2, 11–22]. Among these studies, two randomized studies on radical cystectomy with GDHT showed conflicting results regarding postoperative ileus [2, 18], and another retrospective study reported no benefits of GDHT with regard to postoperative 90-day complications [22]. Because of these inconsistent results, the clinical efficacy of GDHT for radical cystectomy remains controversial.

Thus, in the present study, we aimed to investigate the effects of intraoperative GDHT on the overall postoperative complications in patients who underwent open radical cystectomy. We hypothesized that GDHT during the surgery would have a better effect on clinical outcomes than standard care and compared the incidence of in-hospital postoperative complications, the severity of postoperative complications, the length of hospital stays, and the postoperative laboratory results among the patients.

## Methods

### Ethics

This study was approved by the Institutional Review Board of Seoul National University Hospital (Seoul, Republic of Korea, Approval number: 1712-125-909) and was registered at ClinicalTrials.gov (registration number: NCT03505112, date of registration: 23/04/2018, principal investigator: Jin-Tae Kim). The study was conducted in accordance with the Declaration of Helsinki, and written informed consent was obtained from all the patients. All data were collected at the Seoul National University Hospital between April 2018 and October 2021.

### Participants

We evaluated patients (aged > 20 years) who were scheduled for open radical cystectomy and had American Society of Anesthesiologists (ASA) physical status I–III. We excluded patients who had compromised kidney function (estimated glomerular filtration rate [eGFR] < 60 ml/min/1.73m^2^), compromised liver function, heart failure (New York Heart Association class ≥ III), impaired left ventricular function (ejection fraction < 35%), arrhythmias, and coagulopathies.

### Sample size calculation

Our preliminary investigation of the incidence of in-hospital postoperative complications after open radical cystectomy at the Seoul National University Hospital showed that 40% of the patients had one or more complications after surgery during the hospital stay. Assuming that the incidence of postoperative complications can be reduced from 40 to 12% if patients were managed using GDHT, 37 patients were required, with an alpha of 0.05 and a power of 20% for each group. Considering a 10% drop-out rate, 82 patients were required.

### Randomization and blinding

On the day of surgery, patients were randomly assigned to either the group that received GDHT during the surgery (GDHT group) or the group that received standard care at the discretion of the attending anesthesiologists (control group), with 1:1 allocation based on a randomized computer-generated list, consisting of four and six block sizes. The group allocations were sealed in opaque envelopes by an investigator not involved in the study. Because the anesthesiologists in charge of the operating room managed the patients according to group allocation, blinding the attending anesthesiologists was impossible. However, the investigators who evaluated postoperative outcomes and surgeons were blinded to the group allocation.

### Anesthesia protocol

After the patients entered the operating room, standard monitoring was started, including pulse oximetry, noninvasive blood pressure, electrocardiogram, and bispectral index (BIS). Cerebral oxygenation was also monitored by cerebral oximetry (INVOS 5100 C; Somanetics Co., Troy, MI, USA). Anesthesia was induced with propofol (1.5–2 mg/kg) and remifentanil using a target-controlled infusion (3 ng/ml). After the loss of consciousness, rocuronium (0.6 mg/kg) was administered, and endotracheal intubation was performed. Mechanical ventilation was started using the mode of volume-controlled ventilation. Ventilation parameters were initially set to a tidal volume of 8 ml/kg ideal body weight, a fraction of inspired oxygen (FiO_2_) of 0.5, and an inspiratory-to-expiratory time ratio of 1:2. The respiratory rate was adjusted to maintain an end-tidal carbon dioxide (ETCO_2_) of 35–40 mmHg. The BIS was maintained at 40–60.

After anesthesia induction, a radial artery was catheterized and connected to the FloTrac/EV1000 system (Edwards Lifesciences, Irvine, CA, USA) for continuous monitoring of arterial blood pressure. Then, a central venous catheter was inserted into the right internal jugular vein, and central venous pressure (CVP) was monitored. Hemodynamic variables, including cardiac output (CO), cardiac index (CI), stroke volume index (SVI), and stroke volume variation (SVV), were measured every 20 s.

### Intervention protocol

**Fig. 1:**
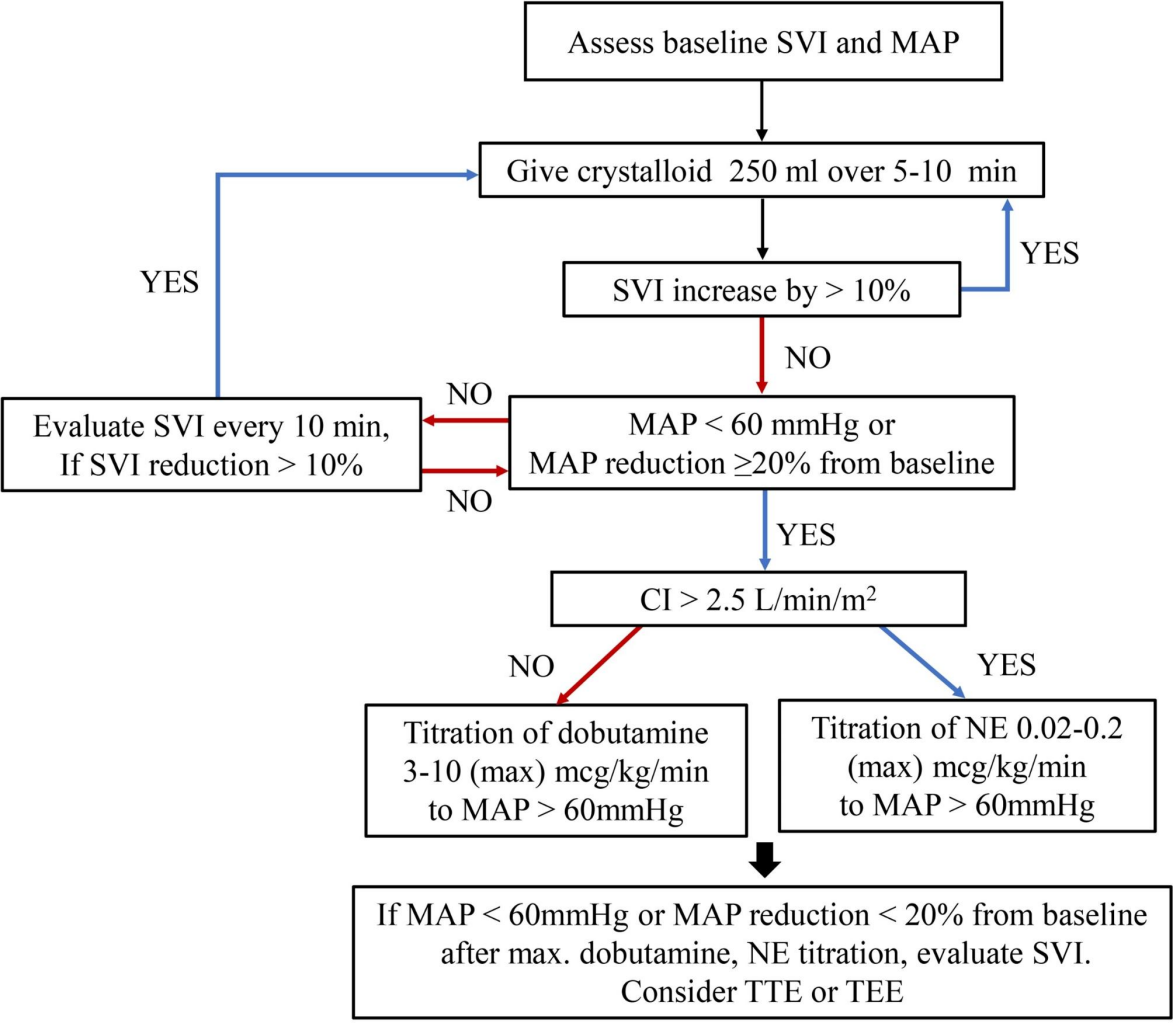
Algorithm for goal-directed hemodynamic therapy SVI stroke volume index, MAP mean arterial pressure, CI cardiac index, NE norepinephrine, TTE transthoracic echocardiography, TEE transesophageal echocardiography

Patients in the control group were managed according to the standard anesthetic techniques at the discretion of the attending anesthesiologists without any specific protocol. The attending anesthesiologists made all decisions regarding the amount and rate of fluid administration and the use of vasoconstrictors and inotropes. Patients in the GDHT group were managed according to the predefined GDHT algorithm (Fig. 1). Baseline SVI and mean arterial pressure (MAP) were measured after anesthesia induction. Next, crystalloid (200–250 ml) was administered over 5–10 min. If the SVI increased by ≥ 10%, an additional 200–250 ml crystalloid was repeatedly infused until the increase in SVI was < 10%. If hypotension (a decrease in MAP of at least 20% from baseline or < 60 mmHg) occurred despite achieving an SVI of < 10% after fluid challenge, the CI was evaluated. If the reduction in the CI (< 2.5 l/min/m^2^) was accompanied by hypotension, dobutamine infusion was started at 3.0 µg/kg/min and adjusted up to a maximum of 10 µg/kg/min so that the CI was higher than 2.5 l/min/m^2^. If the CI did not fall below the threshold, norepinephrine infusion was started at 0.02 µg/kg/min and titrated up to a maximum dose of 0.2 µg/kg/min. If the decrease in MAP persisted despite using a maximum dose of dobutamine or norepinephrine, the SVI was re-evaluated. The SVI and other hemodynamic variables were evaluated every 10 min and managed as needed, according to the algorithm. In the GDHT group, intraoperative fluid administration was maintained at 1 ml/kg/h of crystalloid. Intraoperative blood loss was compensated with a crystalloid infusion at a 1:1 ratio, and transfusion of red blood cells was triggered at the hemoglobin threshold of < 8 g/dl. In both groups, a rescue drug, such as ephedrine at 5 mg or phenylephrine at 30 µg, was allowed for sudden hypotension (systolic blood pressure < 90 mmHg).

### Outcome measures

All data were prospectively collected based on the standard format of our protocol. Patients’ medical history and demographic information, including age, sex, height, weight, ASA physical status classification, underlying disease, and history of any intraperitoneal surgery within the last 5 years, were collected. The following intraoperative and postoperative data were also recorded: type of urinary diversion, operation duration, anesthesia duration, intraoperative and postoperative fluid intake, amount of transfusion, estimated blood loss, intraoperative use of inotropes/vasopressors, and durations of hospital and intensive care unit (ICU) stays. We recorded eGFR, serum creatinine, and C-reactive protein (CRP) on postoperative day (POD) 1, and any changes in these parameters during the hospital stay were also recorded.

The primary outcome was the incidence of a composite of in-hospital postoperative complications. Postoperative complications included seven categories according to the organ system: gastrointestinal, infectious, wound-related (wound dehiscence), cardiac, thromboembolic, genitourinary, and neurologic complications. These complications were assessed according to the Clavien-Dindo classification for radical cystectomy [5, 23]. The complications were independently evaluated by two investigators (HY and DHK) and were confirmed after unanimous agreement was reached. The secondary outcomes were the incidence of each complication in seven categories, duration of hospital stay, duration of ICU stay, grade of complication based on the Clavien-Dindo classification, postoperative laboratory results (eGFR, serum creatinine, and CRP) at POD 1, and changes in these postoperative laboratory parameters during the hospital stay.

### Statistical analysis

The primary analysis used a per-protocol analysis to focus on the efficacy of GDHT, including only patients who strictly followed the protocol. Intention-to-treat analysis was also conducted as a sensitivity analysis to provide insight into the effectiveness of GDHT in diverse clinical settings. Data are presented as mean ± standard deviation, median (interquartile range), or number and percentage. The normality of the distribution of continuous variables was tested using the Kolmogorov–Smirnov test. Continuous variables were analyzed using the Student’s *t*-test or Mann–Whitney *U* test, depending on the data distribution. Categorical data were compared using Fisher’s exact test or the chi-square test. Changes in postoperative eGFR, serum creatinine, and CRP levels were analyzed using a linear mixed-effects model. All statistical analyses were performed using R software (Version 4.0.5, Development Core Team, Vienna, Austria). Results with *P* < 0.05 were considered statistically significant.

## Results

**Fig. 2:**
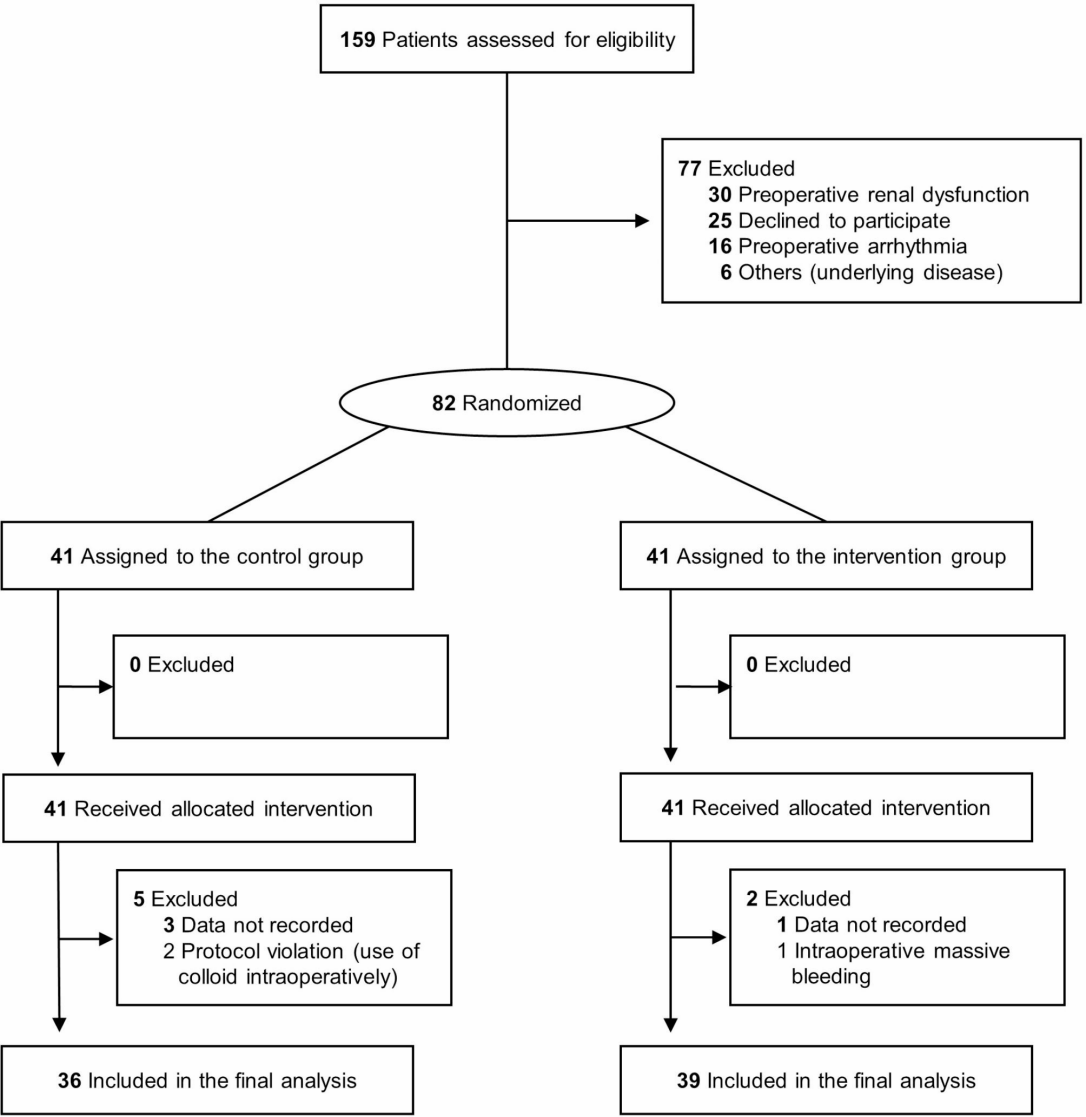
Study flowchart

Among the 159 eligible patients, 77 patients were excluded due to preoperative renal dysfunction, patient refusal, arrhythmia, and other reasons (Fig. 2). During the study period, seven patients had to be excluded: one for intraoperative massive bleeding, two for violations of the protocol regarding intraoperative colloid administration, and four for missing intraoperative data. Patient demographic and baseline characteristics are presented in Table 1.

**Table 1:** Demographic and baseline medical status

Variables	GDHT (N = 39)	Control (N = 36)	Standardized Mean Difference	P-value
Age (years)	66.4 ± 8.5	69.3 ± 7.5	0.361	0.124
Sex, n (%)			0.195	0.565
Male	27 (69.2)	28 (77.8)		
Female	12 (30.8)	8 (22.2)		
ASA classification, n (%)			0.176	0.706
1	4 (10.3)	2 (5.3)		
2	29 (74.4)	27 (75.0)		
3	6 (15.4)	7 (19.4)		
Comorbidities, n (%)				
Hypertension	22 (56.4)	20 (55.6)	0.017	0.999
Diabetes mellitus	7 (17.9)	10 (27.8)	0.232	0.459
Stroke	3 (7.7)	3 (8.3)	0.023	0.999
Cardiac disease	3 (7.7)	4 (11.1)	0.116	0.911
Chronic kidney disease	5 (12.8)	7 (19.4)	0.178	0.641
COPD	2 (5.1)	5 (13.9)	0.298	0.365
Asthma	1 (2.6)	0 (0.0)	0.226	0.999
History of abdominal surgery within 5 years, n (%)	5 (12.8)	3 (8.3)	0.144	0.799
Preoperative C-reactive protein (mg/dl)^*^	0.1 (0.0–0.2)	0.1 (0.1–0.4)	0.401	0.100

Data are expressed as mean ± standard deviation, number (percentage), or median (interquartile range). *This was obtained from 72 patients (37 patients for the GDHT group and 35 patients for the control group, respectively)

GDHT: goal-directed hemodynamic therapy, ASA: American Society of Anesthesiologists, COPD: chronic obstructive pulmonary disease

Table 2 presents the intraoperative characteristics of the patients: the total infused volume of crystalloid was comparable between the two groups (2700 [2175–3250] vs. 2900 [1950–3700] ml, median difference [95% CI], -200 [-875 to 825], *P* = 0.714). Regarding intraoperative hypotension, the areas under the curve for each blood pressure threshold were comparable between the two groups. There were no significant differences in fluid intake and output during the postoperative periods until POD 3 (Table 3).

**Table 2:** Comparisons of intraoperative characteristics between two groups

Variables	GDHT (N = 39)	Control (N = 36)	Risk, median, or mean difference (95% CI)	*P*-value
Type of diversion, n (%)				0.999
Ileal conduit	8 (20.5)	7 (19.4)	0.01 (-0.17 to 0.19)	
Neobladder	31 (79.5)	29 (80.6)	-0.01 (-0.19 to 0.17)	
Duration of surgery (min)	225.0 (195.0–250.0)	227.5 (202.5–260.0)	-2.5 (-30.0 to 12.5)	0.339
Duration of anesthesia (min)	260.0 (237.5–302.5)	270.0 (250.0–307.5)	-10.0 (-35.0 to 10.0)	0.201
Anesthetic agents				0.218
Sevoflurane	9 (23.1)	14 (38.9)	-0.16 (-0.37 to 0.05)	
Desflurane	30 (76.9)	22 (61.1)	0.16 (-0.05 to 0.37)	
Total crystalloid administered (ml)	2700.0 (2175.0–3250.0)	2900.0 (1950.0–3700.0)	-200.0 (-875.0 to 825.0)	0.714
Estimated blood loss (ml)	700.0 (500.0–1000.0)	700.0 (450.0–1250.0)	0 (-350 to 350)	0.671
RBC transfusion (pack)	0.2 ± 0.5	0.3 ± 0.8	-0.15 (-0.47 to 0.16)	0.330
Medications				
Use of norepinephrine, n (%)	5 (12.8)	1 (2.8)	0.10 (-0.02 to 0.22)	0.240
Number of rescue drugs administered, n	5.0 (3.0–8.5)	6.0 (3.5–9.0)	-1.0 (-5.0 to 1.0)	0.166
Amount of ephedrine (mg)	10.0 (5.0–20.0)	17.5 (5.0–30.0)	-7.5 (-15.0 to 2.5)	0.171
Amount of phenylephrine (µg)	0.0 (0.0–65.0)	30.0 (0.0–140.0)	-30.0 (-75.0 to 20.0)	0.131
Amount of remifentanil (µg)	1200.0 (1000.0–1481.5)	1450.0 (1050.0–2000.0)	-250.0 (-635.5 to 100.0)	0.027
Area under MAP (mmHg * min)				
< 65 mmHg	48.0 (14.5–85.5)	56.5 (19.0–94.5)	-8.5 (-36.0 to 31.0)	0.758
< 60 mmHg	5.0 (0.0–46.5)	9.2 (0.0–33.5)	-4.3 (-17.5 to 12.0)	0.913
< 55 mmHg	0.0 (0.0–1.5)	0.0 (0.0–11.0)	0.0 (0.0 to 0.0)	0.664
Extubation in ICU, n (%)	1 (2.6)	0 (0.0)	0.03 (-0.02 to 0.08)	0.999

Data are expressed as number (percentage), median (interquartile range), or mean ± standard deviation

GDHT: goal-directed hemodynamic therapy, CI: confidence interval, RBC: red blood cell, MAP: mean arterial pressure, ICU: intensive care unit

**Table 3:** Comparisons of postoperative fluid balance between two groups

Variables	GDHT (N = 39)	Control (N = 36)	Median difference (95% CI)	*P*-value
Intake (ml)				
Postoperative day 0	1350.0 (925.0–1575.0)	1100.0 (950.0–1351.0)	250.0 (-3.0 to 400.0)	0.062
Postoperative day 1	3250.0 (2971.0–3590.0)	3290.0 (2856.0–3820.0)	-40.0 (-425.0 to 336.5)	0.707
Postoperative day 2	3355.0 (2946.0–3705.0)	3200.0 (2831.0–3790.0)	155.0 (-179.0 to 433.0)	0.535
Postoperative day 3	3250.0 (3090.0–3701.0)	3390.0 (2847.5–3675.0)	-140.0 (-300.0 to 289.0)	0.869
**Output (ml)**				
Postoperative day 0	1302.0 (968.5–1631.5)	1120.0 (767.5–1430.0)	182.0 (-145.5 to 539.0)	0.090
Postoperative day 1	2450.0 (2015.5–2815.5)	2229.5 (1986.0–2701.5)	220.5 (-208.0 to 468.0)	0.413
Postoperative day 2	2295.0 (2082.5–2882.0)	2262.0 (1789.5–2842.5)	33.0 (-291.5 to 538.0)	0.306
Postoperative day 3	2610.0 (2261.5–3078.0)	2267.5 (1990.5–2950.0)	342.5 (-93.0 to 687.0)	0.176

Data are expressed as median (interquartile range)

GDHT: goal-directed hemodynamic therapy, CI: confidence interval

**Fig. 3:**
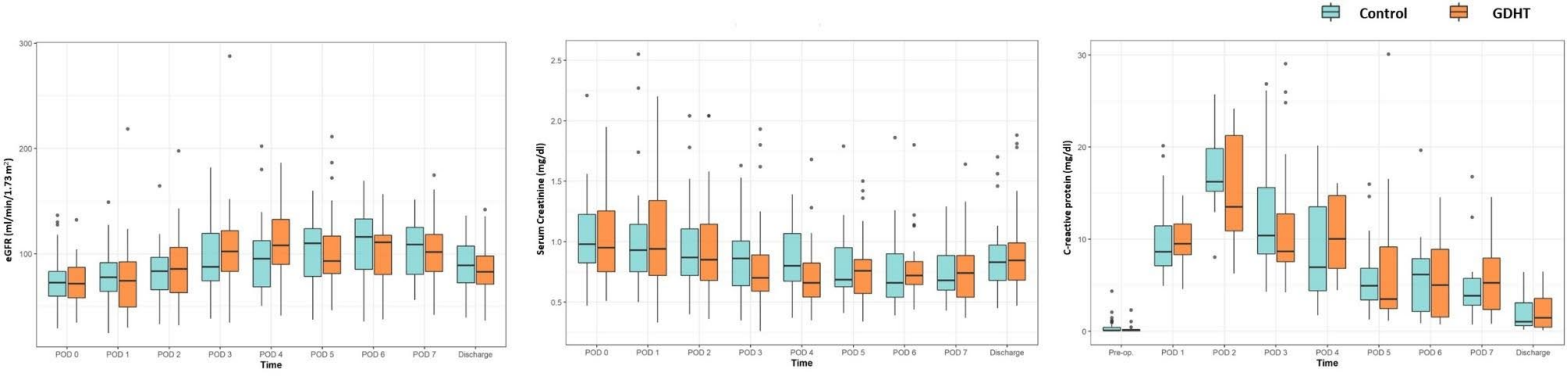
Changes in postoperative laboratory results. (**A**) Estimated glomerular filtration rate, (**B**) Serum creatinine, (**C**) C-reactive protein. eGFR: estimated glomerular filtration rate, GDHT: goal-directed hemodynamic therapy, POD: postoperative day

Regarding the primary outcome, there was no significant difference in the incidence of a composite of in-hospital postoperative complications (28/39 [71.8%] vs. 30/36 [83.3%], risk difference [95% CI], -0.12 [-0.30 to 0.07], *P* = 0.359, Table 4). In addition, the incidence of each of the seven major postoperative complications did not differ significantly between the two groups. The incidence of infectious complications was the highest, followed by genitourinary and wound-related complications. The total duration of hospital stay was comparable between the groups (26.0 [18.0 to 32.0] vs. 24.5 [19.0 to 30.0] days, median difference [95% CI], 1.5 [-5.0 to 5.0], *P* = 0.903). According to the Clavien-Dindo classification, the grade of postoperative complications at POD 1 did not show a statistically significant difference between the two groups (Table 5). The results of the linear mixed-effect analysis showed that the changes in postoperative eGFR, serum creatinine, and CRP during the hospital stay did not differ significantly between the two groups (Fig. 3). In the sensitivity analysis using an intention-to-treat analysis, no significant differences were observed in either primary or secondary outcomes, aligning with the results of the per-protocol analysis (Supplementary Tables S1, 2, 3, 4, and 5).

**Table 4:** Comparisons of postoperative complications and clinical outcomes between two groups

Variables	GDHT (N = 39)	Control (N = 36)	Risk or median difference (95% CI)	P-value
**Total complication, n (%)**	28 (71.8)	30 (83.3)	-0.12 (-0.30 to 0.07)	0.359
**Gastrointestinal complications, n (%)**	6 (15.4)	7 (19.4)	-0.04 (-0.21 to 0.13)	0.874
Ileus	1 (2.6)	1 (2.8)	-0.00 (-0.08 to 0.07)	0.999
Constipation	5 (12.8)	5 (13.9)	-0.01 (-0.16 to 0.14)	0.999
Gastric ulcer	0 (0.0)	0 (0.0)	0.0 (0.0 to 0.0)	NA
Anastomotic bowel leak	1 (2.6)	1 (2.8)	-0.00 (-0.08 to 0.07)	0.999
**Infectious complications, n (%)**	25 (64.1)	19 (52.8)	0.11 (-0.11 to 0.34)	0.447
Urinary tract infection	22 (56.4)	19 (52.8)	0.04 (-0.19 to 0.26)	0.933
Sepsis	9 (23.1)	8 (22.2)	0.01 (-0.18 to 0.20)	0.999
Pneumonia	1 (2.6)	0 (0.0)	0.03 (-0.02 to 0.08)	0.999
Wound infection	4 (10.3)	1 (2.8)	0.07 (-0.03 to 0.18)	0.404
**Wound dehiscence, n (%)**	6 (15.4)	9 (25.0)	-0.10 (-0.28 to 0.09)	0.453
**Cardiac complications, n (%)**	1 (2.6)	4 (11.1)	-0.09 (-0.20 to 0.03)	0.308
Myocardial infarction	1 (2.6)	2 (5.6)	-0.03 (-0.12 to 0.06)	0.944
Arrhythmia	0 (0.0)	2 (5.6)	-0.06 (-0.13 to 0.02)	0.439
Congestive heart failure and pulmonary edema	0 (0.0)	2 (5.6)	-0.06 (-0.13 to 0.02)	0.439
Transient BNP increase	0 (0.0)	1 (2.8)	-0.03 (-0.08 to 0.03)	0.968
**Thromboembolic complications, n (%)**	0 (0.0)	0 (0.0)	0.0 (0.0 to 0.0)	NA
**Genitourinary complications, n (%)**	11 (28.2)	13 (36.1)	-0.08 (-0.29 to 0.13)	0.627
Renal dysfunction	8 (20.5)	7 (19.4)	0.01 (-0.17 to 0.19)	0.999
Renal failure	0 (0.0)	0 (0.0)	0.0 (0.0 to 0.0)	NA
Urinary leakage	6 (15.4)	10 (27.8)	-0.12 (-0.31 to 0.06)	0.305
**Neurologic complications, n (%)**	3 (7.7)	1 (2.8)	0.05 (-0.05 to 0.15)	0.666
**Other complications, n (%)**				
PCD insertion	2 (5.1)	6 (16.7)	-0.12 (-0.26 to 0.02)	0.214
PCN insertion	6 (15.4)	8 (22.2)	-0.07 (-0.25 to 0.11)	0.644
Deep vein thrombosis	1 (2.6)	2 (5.6)	-0.03 (-0.12 to 0.06)	0.944
Total length of hospital stays (days)	26.0 (18.0–32.0)	24.5 (19.0–30.0)	1.5 (-5.0 to 5.0)	0.903
ICU length of stays (days)	0 (0–0)	0 (0–0)	0.0 (0.0 to 0.0)	0.899

Data are expressed as numbers (percentages) or median (interquartile range)

GDHT: goal-directed hemodynamic therapy, CI: confidence interval, NA: not applicable, BNP: brain natriuretic peptide, PCD: percutaneous catheter drainage, PCN: percutaneous nephrostomy, ICU: intensive care unit

**Table 5:** Grades of postoperative complications by the Clavien-Dindo classification

Grades	GDHT (N = 39)	Control (N = 36)	Risk difference (95% CI)	*P*-value
Grade I, n (%)	5 (12.8)	6 (16.7)	-0.04 (-0.20 to 0.12)	0.886
Grade II, n (%)	18 (46.2)	9 (25.0)	0.21 (0.00 to 0.42)	0.096
Grade III, n (%)	11 (28.2)	15 (41.7)	-0.13 (-0.35 to 0.08)	0.327
IIIa	10 (25.6)	14 (38.9)	-0.13 (-0.34 to 0.08)	0.327
IIIb	1 (2.6)	1 (2.8)	-0.00 (-0.08 to 0.07)	0.999
Grade IV, n (%)	0 (0.0)	1 (2.8)	-0.03 (-0.08 to 0.03)	0.480

Data are expressed as numbers (percentages)

GDHT: goal-directed hemodynamic therapy, CI: confidence interval

## Discussion

This study investigated the effect of intraoperative GDHT on postoperative complications in patients who underwent open radical cystectomy. The intervention and control groups showed no significant difference in the incidence of overall complications during the hospital stay. There were also no significant differences in the incidence of each of the seven complications, duration of hospital stays, and the grade of complications. Changes in postoperative eGFR, serum creatinine, and CRP were comparable between the two groups.

Radical cystectomy is the standard therapy for localized bladder cancer with muscle invasion [24]. As the procedure of radical cystectomy involves several adjacent organs, postoperative morbidity and mortality are high [25]. Early complications in open radical cystectomy, defined as complications occurring within postoperative 30 days, have been reported to occur at rates ranging from 39 to 96%, depending on the defining criteria and differences in the reporting periods [3, 5, 18, 25, 26]. These issues make it difficult to compare the estimated postoperative complication rates between existing studies directly. In addition, fluid management in open radical cystectomy may be complicated due to the prolonged duration of surgery, lack of urine measurement, and risk of intraoperative bleeding [22]. A previous study reported the beneficial effects of restrictive fluid administration on postoperative complications and length of hospital stay [23]. However, another retrospective study revealed an association between an increased risk of acute kidney injury and restrictive fluid management [27]. Therefore, fluid and hemodynamic management in open radical cystectomy needs to be optimized through advanced intraoperative monitoring.

In the present study, GDHT did not lead to improved postoperative outcomes. Several reasons might explain this result. First, the two groups had no significant difference in the amount of fluid administered during surgery. Although the amount of fluid administration during surgery was low in the GDHT group, the difference between the groups was not statistically significant. The changes in the trends for administering less fluid during surgery and improvements in perioperative care due to the adoption of the enhanced recovery after surgery (ERAS) protocol may have affected these results [28, 29]. Preoperative dehydration has been rarely observed since the widespread application of the ERAS protocol. Recent improvements in perioperative care over the years can also be inferred from two randomized controlled trials evaluating GDHT, which showed a difference in outcome in open radical cystectomy [2, 18]. They revealed contradictory results regarding postoperative ileus; the older study showed favorable results, while the recent one showed no beneficial effects, consistent with our results. Second, the GDHT algorithm was applied only during surgery, not during the postoperative period, and postoperative care was performed at the discretion of attending clinicians in both groups. Third, according to previous studies, high-risk patients may benefit more from GDHT than non-high-risk patients [10, 30]. However, as most of our patients had ASA physical status II, the benefit from GDHT for our patients may have been less than that for high-risk patients. Since we included patients with a relatively less compromised health status than the patient groups enrolled in previous studies, there were no significant differences in the amount of fluid administered and the use of inotropic or vasoactive medications between the intervention and control groups, and the benefits of intraoperative GDHT could not be demonstrated. Lastly, we evaluated the patients’ hemodynamic status every 10 min; however, a shorter observation time may have presented a more accurate picture of the patient’s hemodynamic status.

Previous studies have reported a U-shaped association between fluid administration during surgery and mortality, indicating that too much or too little intraoperative fluid administration may harm the patients [31, 32]. However, wide variability in fluid administration levels has been reported because intraoperative fluid management is usually at the discretion of treating clinicians [33]. The effect of GDHT on postoperative outcomes has been examined in various studies with inconsistent results [2, 11, 13, 15, 16, 19, 20]; while some studies reported the benefits of GDHT [2, 16], others reported no such benefits [11, 13, 15, 18, 19]. Conflicting results have also been reported in meta-analyses [21, 34–36]. This phenomenon may be attributed to the heterogeneity of the study designs, GDHT algorithms, definitions of primary outcomes, and study durations. Therefore, these results should be interpreted with caution, considering the quality of the evidence.

Our study had some limitations that need to be addressed. First, the sample size was calculated based on the total complication rate of 40% calculated in the preliminary investigation, but the actual complication rate in our study was much higher. This discrepancy may be attributed to the retrospective nature of the pilot study, which likely had missing data on postoperative complications. Second, since the accuracy and reliability of SVI and CI, used as indicators of the patient’s hemodynamic status in the GDHT protocol and obtained by arterial pulse contour analysis, are limited, this limitation might have influenced our results. Third, we did not include an assessment of baseline hemodynamic status at the awake state, which may raise uncertainty about the accuracy of post-induction assessment reflecting the individual patient’s baseline requirement. Fourth, we excluded patients with severe systemic diseases to minimize their potential influence on the effect of GDHT. However, these exclusion criteria also led to the omission of high-risk patients, thereby limiting the generalizability of our study. Hence, further large-scale randomized controlled trials are needed to confirm the benefits of individualized hemodynamic management in high-risk patients undergoing radical cystectomy. Fifth, due to an overly optimistic setting of the effects of GDHT on reducing postoperative complications, our study could be underpowered to detect a significant difference in the primary outcome. Sixth, the involvement of multiple surgeons and changes in anesthesia practice during the study period may have introduced confounding factors to our results. Lastly, we chose a per-protocol analysis to gauge the efficacy of GDHT more accurately. However, we acknowledge that this analysis could introduce biases and may not represent real-world practice despite aligning with the intention-to-treat analysis.

## Conclusions

In summary, we did not find any benefit of GDHT in terms of in-hospital postoperative complications during the hospital stay in the patients who underwent open radical cystectomy. Future research should focus on developing multi-disciplinary, individualized protocols and reliable hemodynamic indicators that reflect the paradigm shift in perioperative patient management.

### Electronic supplementary material

Below is the link to the electronic supplementary material.

Supplementary Material 1

Supplementary Material 2

Supplementary Material 3

Supplementary Material 4

Supplementary Material 5

## Data Availability

The datasets used and/or analysed during the current study are available from the corresponding author on reasonable request.

## Abbreviations

GDHT: Goal-directed hemodynamic therapy
ASA: American Society of Anesthesiologists
eGFR: Estimated glomerular filtration rate
BIS: Bispectral index
FiO_2_: Fraction of inspired oxygen
ETCO_2_: End-tidal carbon dioxide
CVP: Central venous pressure
CO: Cardiac output
CI: Cardiac index
SVI: Stroke volume index
SVV: stroke volume variation
MAP: Mean arterial pressure
ICU: Intensive care unit
CRP: C-reactive protein
POD: Postoperative day
ERAS: Enhanced recovery after surgery
