# Supplementary material for: The effect of goal-directed hemodynamic therapy on clinical outcomes in patients undergoing radical cystectomy: a randomized controlled trial
Source: BMC Anesthesiol. 2023 Oct 9;23:339. doi: 10.1186/s12871-023-02285-9 (PMC10561433; doi:10.1186/s12871-023-02285-9)
Supplement: Supplementary file 1 — Supplementary Material 1 [file 12871_2023_2285_MOESM1_ESM.docx]

| **Variables** | **GDHT**  **(N = 41)** | **Control**  **(N = 41)** | **Standardized Mean Difference** | **P-value** |
| --- | --- | --- | --- | --- |
| Age (years) | 66.8 ± 8.6 | 69.2 ± 7.1 | 0.303 | 0.174 |
| Sex, n (%) |  |  | 0.229 | 0.440 |
| Male | 29 (70.7) | 33 (80.5) |  |  |
| Female | 12 (29.3) | 8 (19.2) |  |  |
| ASA classification, n (%) |  |  | 0.096 | 0.693 |
| 1 | 4 (9.8) | 2 (4.9) |  |  |
| 2 | 29 (70.7) | 31 (75.6) |  |  |
| 3 | 8 (19.5) | 8 (19.5) |  |  |
| Comorbidities, n (%) |  |  |  |  |
| Hypertension | 23 (56.1) | 24 (58.5) | 0.049 | 0.999 |
| Diabetes mellitus | 7 (17.1) | 12 (29.3) | 0.292 | 0.295 |
| Stroke | 3 (7.3) | 4 (9.8) | 0.087 | 0.999 |
| Cardiac disease | 3 (7.3) | 5 (12.2) | 0.165 | 0.710 |
| Chronic kidney disease | 5 (12.2) | 7 (17.1) | 0.138 | 0.755 |
| COPD | 3 (7.3) | 5 (12.2) | 0.165 | 0.710 |
| Asthma | 2 (4.9) | 0 (0.0) | 0.320 | 0.474 |
| History of abdominal surgery within 5 years, n (%) | 5 (12.2) | 3 (7.3) | 0.165 | 0.710 |
| Preoperative C-reactive protein (mg/dl)^*^ | 0.1 (0.0–0.3) | 0.1 (0.1–0.4) | 0.041 | 0.270 |

**Supplementary Table S1** Demographic and baseline medical status (ITT)

Data are expressed as mean ± standard deviation, number (percentage), or median (interquartile range). *This was obtained from 79 patients (39 patients for the GDHT group and 40 patients for the control group, respectively).

GDHT: goal-directed hemodynamic therapy, ASA: American Society of Anesthesiologists, COPD: chronic obstructive pulmonary disease.
