# Supplementary material for: The effect of goal-directed hemodynamic therapy on clinical outcomes in patients undergoing radical cystectomy: a randomized controlled trial
Source: BMC Anesthesiol. 2023 Oct 9;23:339. doi: 10.1186/s12871-023-02285-9 (PMC10561433; doi:10.1186/s12871-023-02285-9)
Supplement: Supplementary file 2 — Supplementary Material 2 [file 12871_2023_2285_MOESM2_ESM.docx]

| **Variables** | **GDHT**  **(N = 41)** | **Control**  **(N = 41)** | **Risk, median, or mean difference (95% CI)** | ***P*-value** |
| --- | --- | --- | --- | --- |
| Type of diversion, n (%) |  |  |  | 0.790 |
| Ileal conduit | 10 (24.4) | 8 (19.5) | 0.05 (-0.13 to 0.23) |  |
| Neobladder | 31 (75.6) | 33 (80.5) | -0.05 (-0.23 to 0.13) |  |
|  |  |  |  |  |
| Duration of surgery (min) | 225.0 (195.0–250.0) | 230.0 (205.0–265.0) | -5.0 (-40.0 to 12.0) | 0.210 |
| Duration of anesthesia (min) | 260.0 (240.0–305.0) | 270.0 (250.0–310.0) | -10.0 (-45.0 to 10.0) | 0.181 |
| Anesthetic agents |  |  |  | 0.225 |
| Sevoflurane | 9 (22.0) | 15 (36.6) | -0.15 (-0.37 to 0.05) |  |
| Desflurane | 32 (78.0) | 26 (63.4) | 0.15 (-0.05 to 0.34) |  |
| Total crystalloid administered (ml) | 2700.0 (2200.0–3300.0) | 2800.0 (1950.0–3700.0) | -100.0 (-900.0 to 800.0) | 0.817 |
| Estimated blood loss (ml) | 700.0 (500.0–1000.0) | 800.0 (460.0–1520.0) | -100 (-450 to 250) | 0.303 |
| RBC transfusion (pack) | 0.3 ± 0.9 | 0.7 ± 1.3 | -0.40 (-0.90 to 0.11) | 0.128 |
|  |  |  |  |  |
| Medications |  |  |  |  |
| Use of norepinephrine, n (%) | 6 (14.6) | 2 (4.9) | 0.10 (-0.03 to 0.22) | 0.264 |
| Number of rescue drugs administered, n | 5.0 (3.0– 9.0) | 7.0 (4.0–9.0) | -2.0 (-4.0 to 1.0) | 0.187 |
| Amount of ephedrine (mg) | 10.0 (5.0–20.0) | 15.0 (5.0–30.0) | -5.0 (-15.0 to 5.0) | 0.271 |
| Amount of phenylephrine (μg) | 0.0 (0.0–80.0) | 40.0 (0.0–160.0) | -40.0 (-120.0 to 10.0) | 0.070 |
| Amount of remifentanil (μg) | 1200.0 (1000.0–1463.0) | 1400.0 (1000.0–2000.0) | -200.0 (-494.0 to 100.0) | 0.049 |
|  |  |  |  |  |
| Area under MAP (mmHg * min) |  |  |  |  |
| < 65 mmHg | 48.0 (17.0–87.0) | 58.0(20.0–99.0) | -8.5 (-36.0 to 31.0) | 0.781 |
| < 60 mmHg | 7.0 (0.0–49.0) | 11.0 (0.0–34.0) | -4.3 (-17.5 to 12.0) | 0.735 |
| < 55 mmHg | 0.0 (0.0–3.0) | 0.0 (0.0–11.0) | 0.0 (0.0 to 0.0) | 0.888 |
|  |  |  |  |  |
| Extubation in ICU, n (%) | 2 (4.9) | 0 (0.0) | 0.03 (-0.02 to 0.08) | 0.474 |

**Supplementary Table S2** Comparisons of intraoperative characteristics between two groups.

Data are expressed as number (percentage), median (interquartile range), or mean ± standard deviation.

GDHT: goal-directed hemodynamic therapy, CI: confidence interval, RBC: red blood cell, MAP: mean arterial pressure, ICU: intensive care unit.
