# Supplementary material for: The effect of goal-directed hemodynamic therapy on clinical outcomes in patients undergoing radical cystectomy: a randomized controlled trial
Source: BMC Anesthesiol. 2023 Oct 9;23:339. doi: 10.1186/s12871-023-02285-9 (PMC10561433; doi:10.1186/s12871-023-02285-9)
Supplement: Supplementary file 3 — Supplementary Material 3 [file 12871_2023_2285_MOESM3_ESM.docx]

| **Variables** | **GDHT (N = 41)** | **Control (N = 41)** | **Median difference (95% CI)** | ***P*-value** |
| --- | --- | --- | --- | --- |
| **Intake (ml)** |  |  |  |  |
| Postoperative day 0 | 1350.0 (900.0–1555.0) | 1102.0 (950.0–1352.0) | 248.0 (-50.0 to 399.0) | 0.115 |
| Postoperative day 1 | 3250.0 (2952.0–3600.0) | 3332.0 (2880.0–3790.0) | -82.0 (-462.0 to 250.0) | 0.568 |
| Postoperative day 2 | 3410.0 (2960.0–3700.0) | 3130.0 (2800.0–3610.0) | 280.0 (-110.0 to 530.0) | 0.317 |
| Postoperative day 3 | 3270.0 (3100.0–3720.0) | 3360.0 (2750.0–3670.0) | -90.0 (-258.0 to 345.0) | 0.490 |
|  |  |  |  |  |
| **Output (ml)** |  |  |  |  |
| Postoperative day 0 | 1302.0 (1002.0–1623.0) | 1150.0 (775.0–1465.0) | 152.0 (-185.0 to 463.0) | 0.199 |
| Postoperative day 1 | 2410.0 (1996.0–2797.0) | 2237.0(1995.0–2778.0) | 173.0 (-280.0 to 417.0) | 0.683 |
| Postoperative day 2 | 2295.0 (2055.0–2884.0) | 2198.0 (1808.0–2772.0) | 97.0 (-314.0 to 577.0) | 0.258 |
| Postoperative day 3 | 2610.0 (2255.0–3108.0) | 2275.0 (2000.0–3030.0) | 335.0 (-78.0 to 687.0) | 0.191 |

**Supplementary Table S3** Comparisons of postoperative fluid balance between two groups

Data are expressed as median (interquartile range).

GDHT: goal-directed hemodynamic therapy, CI: confidence interval.
