# Supplementary material for: The effect of goal-directed hemodynamic therapy on clinical outcomes in patients undergoing radical cystectomy: a randomized controlled trial
Source: BMC Anesthesiol. 2023 Oct 9;23:339. doi: 10.1186/s12871-023-02285-9 (PMC10561433; doi:10.1186/s12871-023-02285-9)
Supplement: Supplementary file 4 — Supplementary Material 4 [file 12871_2023_2285_MOESM4_ESM.docx]

**Supplementary Table S4** Comparisons of postoperative complications and clinical outcomes between two groups

| **Variables** | **GDHT (N = 41)** | **Control (N = 41)** | **Risk or median difference (95% CI)** | **P-value** |
| --- | --- | --- | --- | --- |
| **Total complication, n (%)** | 28 (68.3) | 34 (82.9) | -0.15 (-0.33 to 0.04) | 0.199 |
| **Gastrointestinal complications, n (%)** | 6 (14.6) | 8 (19.5) | -0.05 (-0.21 to 0.11) | 0.769 |
| Ileus | 1 (2.4) | 2 (4.9) | -0.02 (-0.11 to 0.06) | 0.999 |
| Constipation | 5 (12.2) | 5 (12.2) | -0.00 (-0.14 to 0.14) | 0.999 |
| Gastric ulcer | 0 (0.0) | 0 (0.0) | 0.0 (0.0 to 0.0) | NA |
| Anastomotic bowel leak | 1 (2.4) | 2 (4.9) | -0.02 (-0.11 to 0.06) | 0.999 |
|  |  |  |  |  |
| **Infectious complications, n (%)** | 25 (61.0) | 23 (56.1) | 0.05 (-0.16 to 0.26) | 0.823 |
| Urinary tract infection | 22 (53.7) | 23 (56.1) | -0.02 (-0.24 to 0.19) | 0.999 |
| Sepsis | 9 (22.2) | 8 (19.5) | 0.02 (-0.15 to 0.20) | 0.999 |
| Pneumonia | 1 (2.4) | 1 (2.4) | 0.00 (-0.07 to 0.08) | 0.999 |
| Wound infection | 4 (9.8) | 1 (2.4) | 0.07 (-0.03 to 0.18) | 0.404 |
|  |  |  |  |  |
| **Wound dehiscence, n (%)** | 6 (14.6) | 10 (24.4) | -0.10 (-0.27 to 0.07) | 0.403 |
|  |  |  |  |  |
| **Cardiac complications, n (%)** | 1 (2.4) | 4 (9.8) | -0.07 (-0.18 to 0.03) | 0.356 |
| Myocardial infarction | 1 (2.4) | 2 (4.9) | -0.02 (-0.11 to 0.06) | 0.944 |
| Arrhythmia | 0 (0.0) | 2 (4.9) | -0.05 (-0.11 to 0.02) | 0.474 |
| Congestive heart failure and pulmonary edema | 0 (0.0) | 2 (4.9) | -0.05 (-0.11 to 0.02) | 0.474 |
| Transient BNP increase | 0 (0.0) | 3 (7.3) | -0.07 (-0.15 to 0.01) | 0.239 |
|  |  |  |  |  |
| **Thromboembolic complications, n (%)** | 0 (0.0) | 1 (2.4) | -0.02 (-0.07 to 0.02) | 0.999 |
|  |  |  |  |  |
| **Genitourinary complications, n (%)** | 11 (26.8) | 16 (39.0) | -0.12 (-0.32 to 0.08) | 0.347 |
| Renal dysfunction | 8 (19.5) | 7 (17.1) | 0.02 (-0.14 to 0.19) | 0.999 |
| Renal failure | 0 (0.0) | 0 (0.0) | 0.0 (0.0 to 0.0) | NA |
| Urinary leakage | 6 (14.6) | 13 (31.7) | -0.17 (-0.35 to 0.01) | 0.116 |
|  |  |  |  |  |
| **Neurologic complications, n (%)** | 3 (7.3) | 1 (2.4) | 0.05 (-0.04 to 0.14) | 0.608 |
|  |  |  |  |  |
| **Other complications, n (%)** |  |  |  |  |
| PCD insertion | 2 (4.9) | 9 (22.0) | -0.17 (-0.31 to -0.03) | 0.052 |
| PCN insertion | 7 (17.1) | 10 (24.4) | -0.07 (-0.25 to 0.10) | 0.586 |
| Deep vein thrombosis | 1 (2.4) | 2 (4.9) | -0.02 (-0.11 to 0.06) | 0.999 |
|  |  |  |  |  |
| Total length of hospital stays (days) | 24.0 (17.0–31.0) | 28.0 (20.0–32.0) | -4.0 (-7.0 to 4.0) | 0.414 |
| ICU length of stays (days) | 0 (0–0) | 0 (0–0) | 0.0 (0.0 to 0.0) | 0.655 |

Data are expressed as numbers (percentages) or median (interquartile range).

GDHT: goal-directed hemodynamic therapy, CI: confidence interval, NA: not applicable, BNP: brain natriuretic peptide, PCD: percutaneous catheter drainage, PCN: percutaneous nephrostomy, ICU: intensive care unit.
