# Supplementary material for: The effect of goal-directed hemodynamic therapy on clinical outcomes in patients undergoing radical cystectomy: a randomized controlled trial
Source: BMC Anesthesiol. 2023 Oct 9;23:339. doi: 10.1186/s12871-023-02285-9 (PMC10561433; doi:10.1186/s12871-023-02285-9)
Supplement: Supplementary file 5 — Supplementary Material 5 [file 12871_2023_2285_MOESM5_ESM.docx]

| **Grades** | **GDHT (N = 41)** | **Control (N = 41)** | **Risk difference (95% CI)** | ***P*-value** |
| --- | --- | --- | --- | --- |
| Grade Ⅰ, n (%) | 5 (12.2) | 6 (14.6) | -0.02 (-0.17 to 0.12) | 0.999 |
| Grade Ⅱ, n (%) | 18 (43.9) | 10 (24.4) | 0.20 (-0.01 to 0.40) | 0.103 |
| Grade Ⅲ, n (%) | 12 (29.3) | 18 (43.9) | -0.15 (-0.35 to 0.06) | 0.252 |
| Ⅲa | 11 (26.8) | 17 (41.5) | -0.15 (-0.35 to 0.06) | 0.244 |
| Ⅲb | 1 (2.4) | 1 (2.4) | 0.00 (-0.07 to 0.07) | 0.999 |
| Grade Ⅳ, n (%) | 0 (0.0) | 1 (2.4) | -0.02 (-0.07 to 0.02) | 0.999 |

**Table 5** Grades of postoperative complications by the Clavien-Dindo classification

Data are expressed as numbers (percentages).

GDHT: goal-directed hemodynamic therapy, CI: confidence interval.
